# Fatigue Behaviour of 7N01-T4 Aluminium Alloy Welded by Ultrasonic-Assisted Friction Stir Welding

**DOI:** 10.3390/ma13204582

**Published:** 2020-10-14

**Authors:** Zhiqiang Zhang, Changshu He, Ying Li, Jingxun Wei, Menggang Zhai, Su Zhao, Xiang Zhao

**Affiliations:** 1 School of Materials Science and Engineering, Northeastern University, Shenyang 110819, China; lnkdzzq@126.com (Z.Z.); liying3273@163.com (Y.L.); 18978226891@163.com (J.W.); zhaox@mail.neu.edu.cn (X.Z.); 2 Key Laboratory for Anisotropy and Texture of Materials, Northeastern University, Shenyang 110819, China; 3 Ningbo Institute of Materials Technology & Engineering, Chinese Academy of Sciences, Ningbo 315201, China; zhaimgmail@nimte.ac.cn (M.Z.); zhaosu@nimte.ac.cn (S.Z.)

**Keywords:** ultrasonic-assisted friction stir welding, aluminium alloy, fatigue behaviour, microstructure, residual stresses

## Abstract

This study investigates the effects of axial ultrasonic vibration on the microstructure evolution, residual stresses distribution and fatigue fracture behaviour of a 7N01-T4 joint, and demonstrates that ultrasonic vibration can significantly promote the flow of plasticised metals, expand the stirred zone (SZ) width and refine the grain size. The longitudinal residual stresses of the joints are dominant, and the peak longitudinal residual stresses of the thermo-mechanically affected zone (TMAZ) on the advancing side (AS) (TMAZ-AS) in the ultrasonic-assisted friction stir welding (UAFSW) joint are 31.5 MPa lower than those in the friction stir welding (FSW) joint. Compared to that of FSW joints, the fatigue strength of UAFSW joints increases by 20 MPa at 10^7^ cycles (stress ratio of *R* = 0.1). At high-stress levels, crack initiation occurs at the TMAZ-AS, and is mainly attributed to high residual stresses and second-phase particles. At low-stress levels, fatigue cracks are likely to initiate in the transition zone (TZ).

## 1. Introduction

Friction stir welding (FSW) is a revolutionary welding technology invented by The Welding Institute in the 1990s. It is especially suitable for welding aluminium alloys [1,2], and is widely used in aerospace, ships and pressure vessels. Fatigue fracture is the main failure form of welded structural parts. Therefore, researchers mainly focus on the fatigue behaviour and fatigue performance of FSW joints. Generally, the S-N curve and fatigue crack growth behaviour of FSW joints are the effective ways to evaluate the fatigue performance of joints [3,4]. The fatigue performance of FSW joints is mainly related to the surface roughness, micro-defects, stress state and intermetallic compounds. Research on the surface characteristics of the joint found that cracks are prone to occur on the circular arc and the flash on both sides of the joint [5,6]. The kissing bond seriously deteriorates the fatigue performance of the FSW joint [7,8,9]. The double-sided FSW process can effectively avoid the kissing bond formation at the bottom of the joint, thereby improving the fatigue performance of the joint [10]. During the FSW process, the metal in the stirred zone (SZ) experiences high temperature and severe deformation. The uneven microstructure of the micro-zone and the welding temperature gradient lead to large residual stresses around the weld zone [11]. Therefore, when the local yield strength of the joint is lower than the superimposed external and residual stresses of the micro-zone, deformation preferentially occurs at this location, leading to fatigue cracks initiation [12]. The fatigue fracture of heat-treatable aluminium alloy FSW joints generally often occurs in the lowest hardness zone in the heat-affected zone (HAZ) [13]. However, the location of fatigue crack initiation is not entirely determined by the static mechanical properties of the FSW joints. He et al. [14] found that some intermetallic compounds were broken in the thermo-mechanically affected zone (TMAZ) during the intense plastic deformation, which easily became the crack initiation zone. Besel et al. [15] also found that joint hardness was not the only factor affecting the fatigue crack initiation or propagation behaviour; instead, all fatigue cracks were located around the SZ and TMAZ. In addition, the nonuniform deformation near the SZ/TMAZ boundary also induces the formation of fatigue crack initiation [16].

The ultrasonic vibration-assisted plastic deformation process can significantly reduce the flow stress and forming force [17]. In recent years, some researchers have combined ultrasonic vibration with FSW to develop the ultrasonic-assisted friction stir welding (UAFSW) technology. At present, according to the application position of ultrasonic vibration in the FSW process, it can be divided into the following ways: Ultrasonic vibration is applied (1) on the workpiece [18,19,20,21], (2) on the tool in the lateral direction [22,23] and (3) on the tool in the axial direction [24,25]. Regardless of the method of applying ultrasonic vibration, the publicly reported results showed that ultrasonic vibration can promote metal flow in the weld zone, eliminate defects, and reduce welding load. However, studies on the effect of ultrasonic vibration on the FSW joint fatigue behaviour are limited. Although it has been reported that applying ultrasonic vibration on the workpiece can improve the fatigue performance of the 2024 aluminium alloy FSW joint, residual stresses of the joint have not been measured, and the fatigue fracture behaviour of the joint under different stress conditions has not been systematically studied [26]. The 7N01 alloy has been employed in body structures of high-speed trains, and the mechanical properties (especially fatigue properties) of the FSW joints are very important for the reliability and safety of high-speed trains [27]. Our previous studies performed systematic studies on the microstructure evolution and tensile properties of 7N01-T4 aluminium alloy joints by axial ultrasonic vibration-assisted FSW [25]. It was found that the axial ultrasonic vibration can significantly promote the metal flow in the SZ along the thickness direction, the structure is more uniform and dense, and the static mechanical properties of the joint are improved.

Therefore, based on our previous work, this paper focuses on the effect of ultrasonic vibration on the fatigue properties of 7N01-T4 aluminium alloy FSW joints. The fracture behaviour of the joints was systematically analysed from perspectives of the metal flow, microstructure characteristic evolution, and residual stresses distribution characteristics to reveal the fatigue fracture mechanism of 7N01-T4 aluminium alloy UAFSW joints.

## 2. Materials and Methods

**Figure 1:**
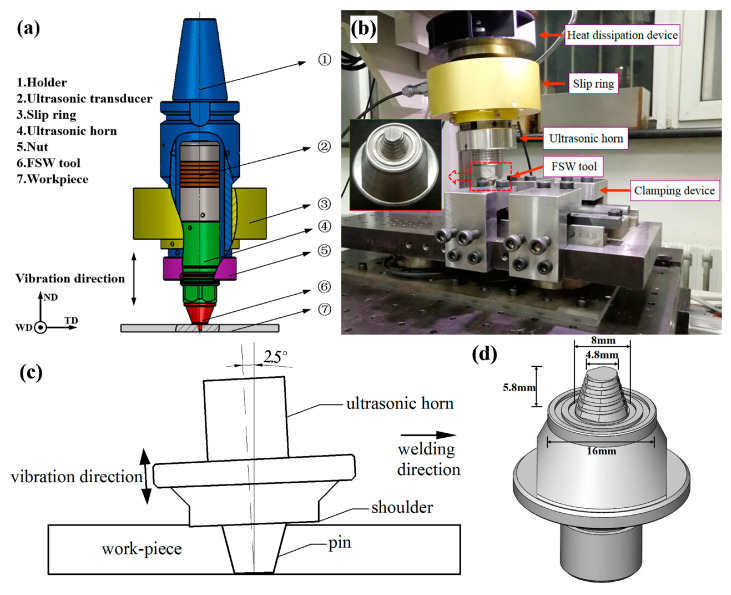
Schematic diagram of the ultrasonic-assisted friction stir welding (UAFSW) system (**a**); experimental device (**b**); UAFSW processing (**c**); and friction stir weld tool (**d**).

**Figure 2:**
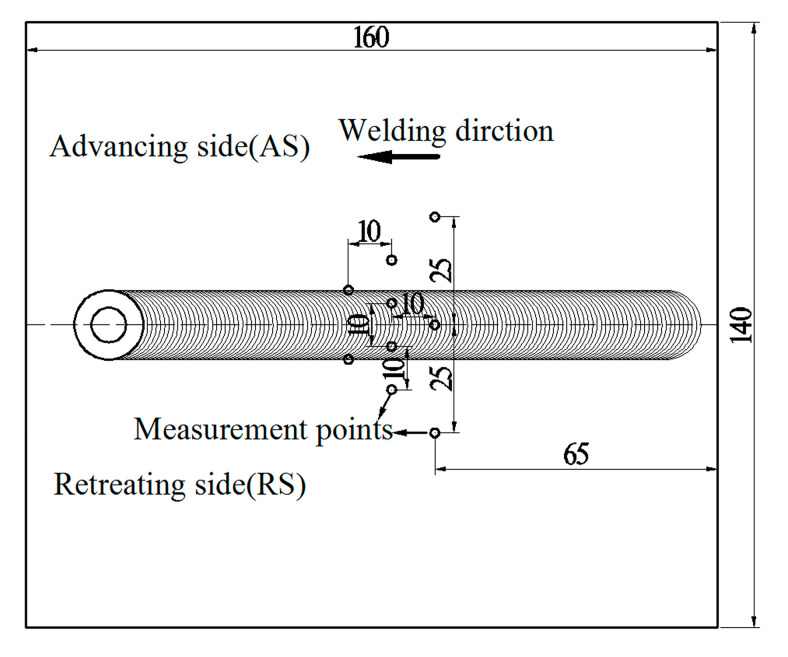
Stress measurement points on the welding specimen (unit: mm).

**Figure 3:**
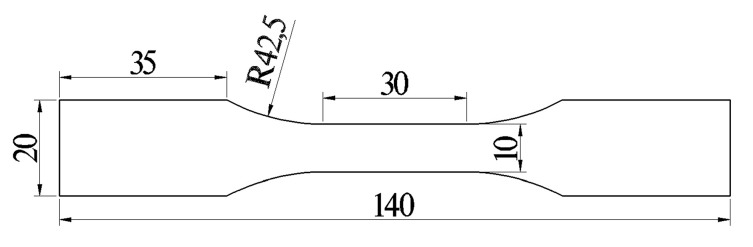
Dimensions of the fatigue test specimen (unit: mm).

In this study, we used 7N01-T4 (Al–4.48Zn–1.03Mg–0.076Cu (wt.%)) aluminium alloy plates that were 160 mm (length) × 70 mm (width) × 6 mm (thickness) to conduct butt FSW and UAFSW experiments. The welding process was performed parallel to the rolling direction of the 7N01-T4 plates. Figure 1a,b depict the schematic diagram of the axial ultrasonic vibration-assisted FSW and the experimental device. The tool and the ultrasonic transducer are integrated. The rotating tool simultaneously performs high-frequency axial vibration; hence, the ultrasonic energy acts on the weld metal with minimum loss (Figure 1c). The ultrasonic vibration system operated with 20 kHz frequency and 10 μm amplitude. Figure 1d presents the welding tool. FSW and UAFSW were performed with the same optimised welding parameters. The welding and rotation speeds of 160 mm/min and 1200 rpm, respectively, were employed. The plunge depth of the shoulder was 0.2 mm. The tool tilt angle was 2.5° with respect to the spindle of the welding machine. After welding, the welding flashes of the surface layer and the ‘semicircular arc pattern’ structure were polished by emery papers. Residual stresses measurements of the FSW and UAFSW joints were performed by means of the strain gauge hole-drilling method, according to the ASTM E837 standard. Special residual stresses drilling equipment and software were selected for the experiment and data analysis. Holes were drilled in 2 mm depth with 1.5 mm diameter. The strain gauge type was BSF120-1.5CA-T (Sigmar, Jinan, China) with 2.08 ± 0.5% sensitivity. The residual stresses distribution around the centre line of the weld was analysed. Figure 2 illustrates the test locations. The cross-section of specimens was polished and finally etched by Keller’s reagent (1% HF + 1.5% HCl + 2.5% HNO_3_ + 95% H_2_O). Subsequently, the microstructure features were observed through optical microscopy (Olympus-GX71, Olympus, Tokyo, Japan). Fatigue tests were conducted at room temperature on a high-frequency fatigue testing machine at a loading frequency range of 90–100 Hz and a stress ratio of 0.1. Fatigue specimen welds were transverse to the stress axis direction of the fatigue testing machine. The specimens were polished prior to the fatigue tests. Figure 3 shows the dimensions of the fatigue test specimen. When the number of cycles exceeded 10^7^, the fatigue stress was defined as the fatigue limit herein. The fatigue fracture locations were counted after the fatigue tests. Fatigue fracture surfaces of the FSW and UAFSW failed specimens were compared and observed using JSM-7001F scanning electron microscopy (JEOL, Tokyo, Japan).

## 3. Results

### 3.1. Microstructure

**Figure 4:**
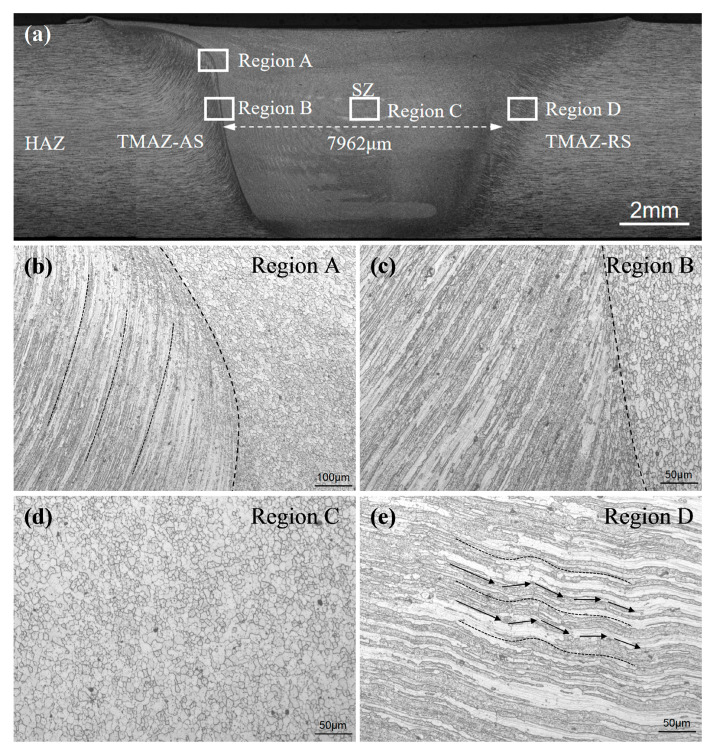
Macrostructure and microstructure of the UAFSW joint: (**a**) Macrostructure; (**b**) stirred zone/thermo-mechanically affected zone (SZ/TMAZ) junction; (**c**) thermo-mechanically affected zone (TMAZ) on the advancing side (TMAZ-AS); (**d**) SZ; and (**e**) TMAZ on the retreating side (TMAZ-RS).

**Figure 5:**
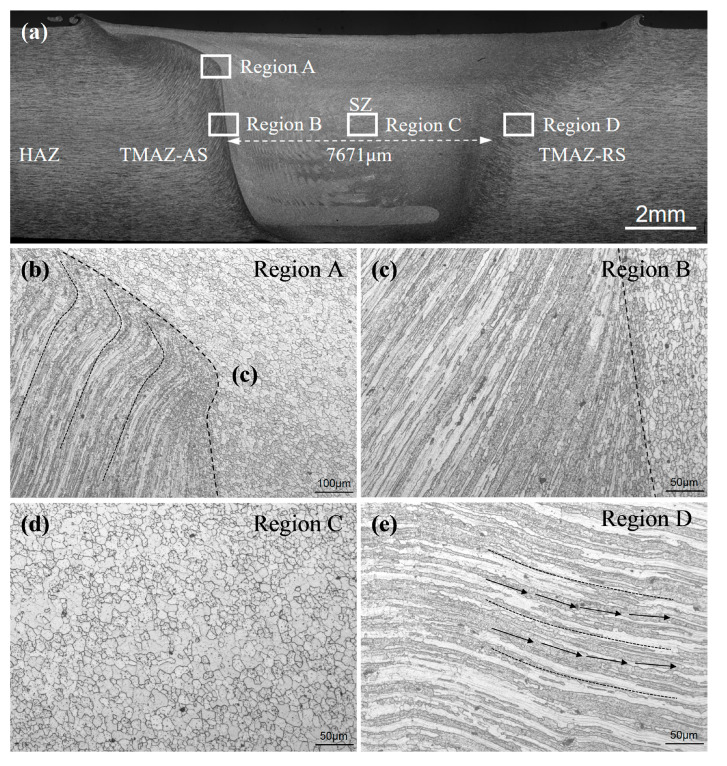
Macrostructure and microstructure of the FSW joint: (**a**) Macrostructure; (**b**) SZ/TMAZ junction; (**c**) TMAZ-AS; (**d**) SZ and (**e**) TMAZ-RS.

Figure 4 and Figure 5 display the macro cross-section and microstructures of the UAFSW and FSW joints, respectively. Except for the base material, the joint was divided into the HAZ, TMAZ and SZ. By comparison, the width of the UAFSW SZ was 7962 μm, which is larger than that of the FSW (i.e., 7671 μm). Note that the junction transition between the SZ and the TMAZ of the UAFSW joint was smooth (Region A, Figure 4), whilst the same position of the FSW joint presented an obvious ‘bump’ feature (Region A, Figure 5). This indicates that ultrasonic vibration reduced the external force required to deform the material, and the flow ability of the metal was improved. The microstructure difference between the FSW and UAFSW joints in the TMAZ on the advancing side (AS) (TMAZ-AS) was not obvious, and presented a large degree of the upward flowing shape (Region B). The microstructure of the SZ was characterised by refined and equiaxed grains (Region C). In the early period of thermal-mechanical deformation, high-density dislocations are introduced, and many sub-grains were formed through dynamic recovery. Sub-grains absorb the dislocations to increase misorientation of the grain boundary, resulting in formation of equiaxed grains [28]. The axial high-frequency vibration induced the ultrasonic energy into the SZ through the tool, which provided a strong instantaneous impact on the metal flow of the SZ. The SZ metal moved violently in the plate thickness direction, and the deformation was greater; a large number of dislocations were tangled, thereby forming dislocation walls and dislocation cells, which promoted the UAFSW joint substructure formation and refined the grains [29]. The grain bending state of the TMAZ on the retreating side (RS) of the FSW and UAFSW joints was different. The deformation grains of the TMAZ-RS in the FSW joint were streamlined. Furthermore, the grains in the UAFSW joint exhibited tortuous distribution characteristics (Region D, Figure 4). The high-frequency axial movement of the tool resulted in the cooperative deformation of the RS metal near the SZ.

### 3.2. Residual Stresses Testing

**Figure 6:**
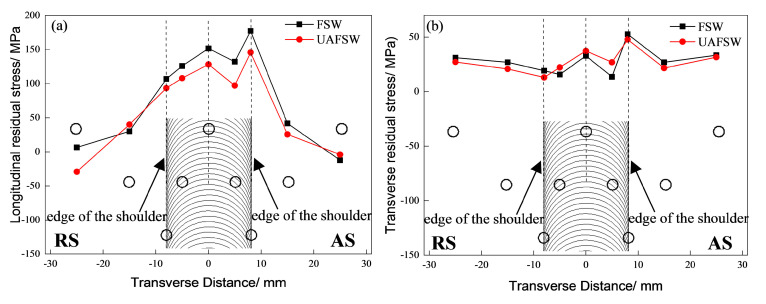
Residual stresses for the FSW and UAFSW joints: (**a**) Longitudinal residual stresses and (**b**) transverse residual stresses.

Figure 6 shows the longitudinal and transverse residual stresses of the FSW and UAFSW joints. The longitudinal residual stresses presented an ‘M’-shaped distribution characteristic. The peak longitudinal residual stresses occurred on the AS of the shoulder edge [30,31,32], and the peak longitudinal residual stresses of the FSW and UAFSW joints at the TMAZ-AS were 177.3 and 145.8 MPa, respectively. Ultrasonic vibration can reduce the longitudinal residual stresses by approximately 17.8%. Residual stresses were mainly related to the temperature and the degree of the metal being stirred. The FSW heat mainly came from the frictional heat generated by the relative sliding between the shoulder and workpiece, in which the metal flow relative velocity at the shoulder edge was largest, thereby resulting in the highest peak temperature. In addition, the flow direction of the material on the AS was the same as the tool welding direction, and the material was subjected to longitudinal tensile stresses, whereas the material was subjected to longitudinal compressive stresses on the RS. The temperature fields of the AS and the RS are superimposed on different stress types, reflecting the asymmetric distribution of the longitudinal residual stresses in the joint. The horn drive tool vibrated in the axial direction, and the shoulder and the workpiece were in an alternating ‘contact-separation’ state, which resulted in the reduced friction and heat generation of the shoulder edge and the workpiece. The axial ultrasonic vibration can effectively enhance the metal flow ability and decrease the deformation stress of the metal; thus, the longitudinal residual stresses of the joint were reduced. The location away from the weld zone was not subjected to the stirring effect of the pin, was less affected by the welding thermal cycle, and was finally converted into compressive stress to maintain the stress balance. The transverse residual stresses of the joint were relatively low (Figure 6b), and those of both the FSW and UAFSW joints were 10–50 MPa.

### 3.3. Fatigue Strength

**Figure 7:**
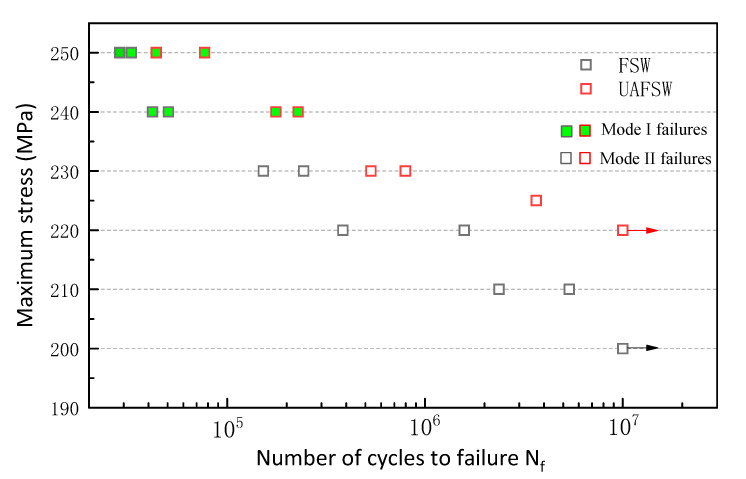
S–N diagram of the FSW and UAFSW specimens.

**Figure 8:**
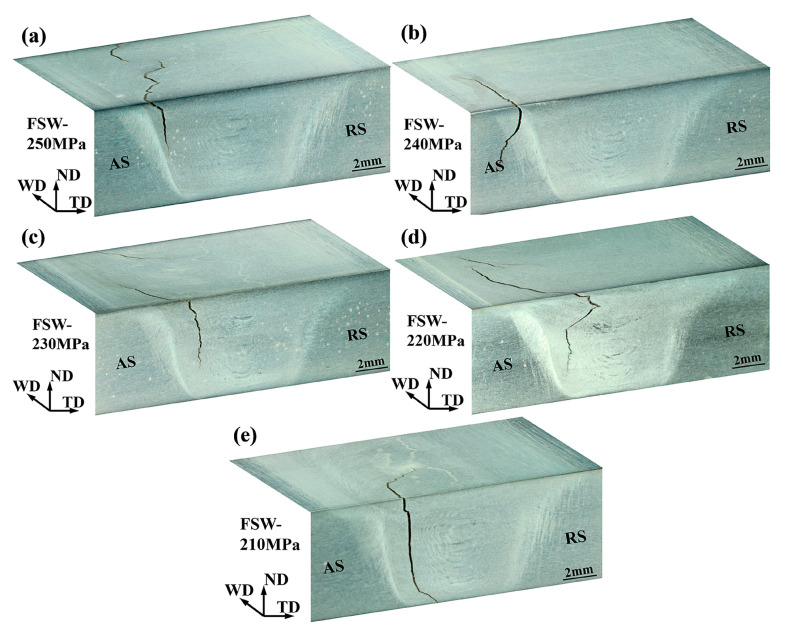
Fracture locations of the FSW fatigue specimens at different maximum stresses of 250 (**a**); 240 (**b**); 230 (**c**); 220 (**d**) and 210 MPa (**e**).

**Figure 9:**
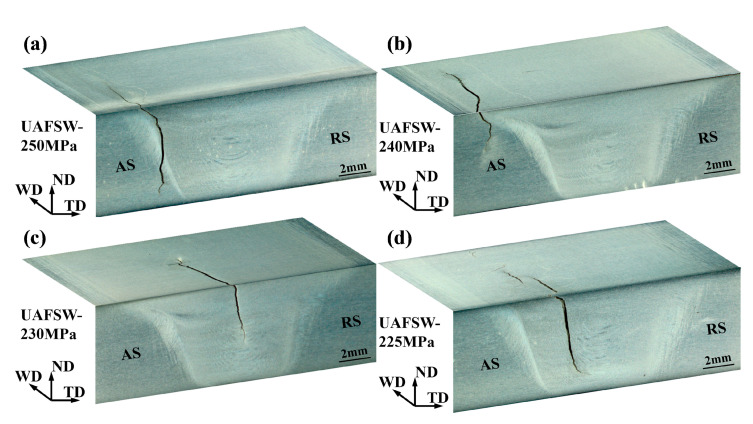
Fracture locations of the UAFSW fatigue specimens at different maximum stresses of 250 (**a**); 240 (**b**); 230 (**c**) and 225 MPa (**d**).

Figure 7 depicts the S–N diagram of the FSW and UAFSW specimens. FSW specimens were tested at the selected stress level of 250–200 MPa, and UAFSW specimens were tested at 250–220 MPa. At least two fatigue specimens were tested at each selected stress level where fracture failure occurred (except for the UAFSW specimen at 225 MPa). The specimens that did not fail before 1 × 10^7^ cycles are represented by the arrows. For the FSW joints, the fatigue limit of 10^7^ cycles was 200 MPa, whereas that of the UAFSW sample was greatly increased to approximately 220 MPa. The fatigue limit of the FSW specimen was lower than that of the UAFSW specimen, although the tensile strength of the two joints was comparable [25]. The UAFSW exhibited a longer fatigue life than the FSW at the same stress amplitude. It is worth noting that there are two failure modes according to the location of the initial cracks of the fatigue specimens in Figure 7 (mode I failures and mode II failures). The fracture locations of the FSW and UAFSW specimens are shown in Figure 8 and Figure 9. The FSW and UAFSW specimens showed a fatigue fracture near the TMAZ-AS under high-stress levels (i.e., 250–240 MPa), which belongs to mode I failures. By contrast, at low-stress levels of 230–210 MPa, both joints showed a fracture at the SZ, which are mode II failures. The difference in the fatigue limit between the UAFSW specimen and FSW specimen and the two modes of failures may be attributed to the microstructure and stress state of the joint. This requires further discussion on the initiation mechanism of fatigue cracks.

### 3.4. Fatigue Fracture Behaviour

**Figure 10:**
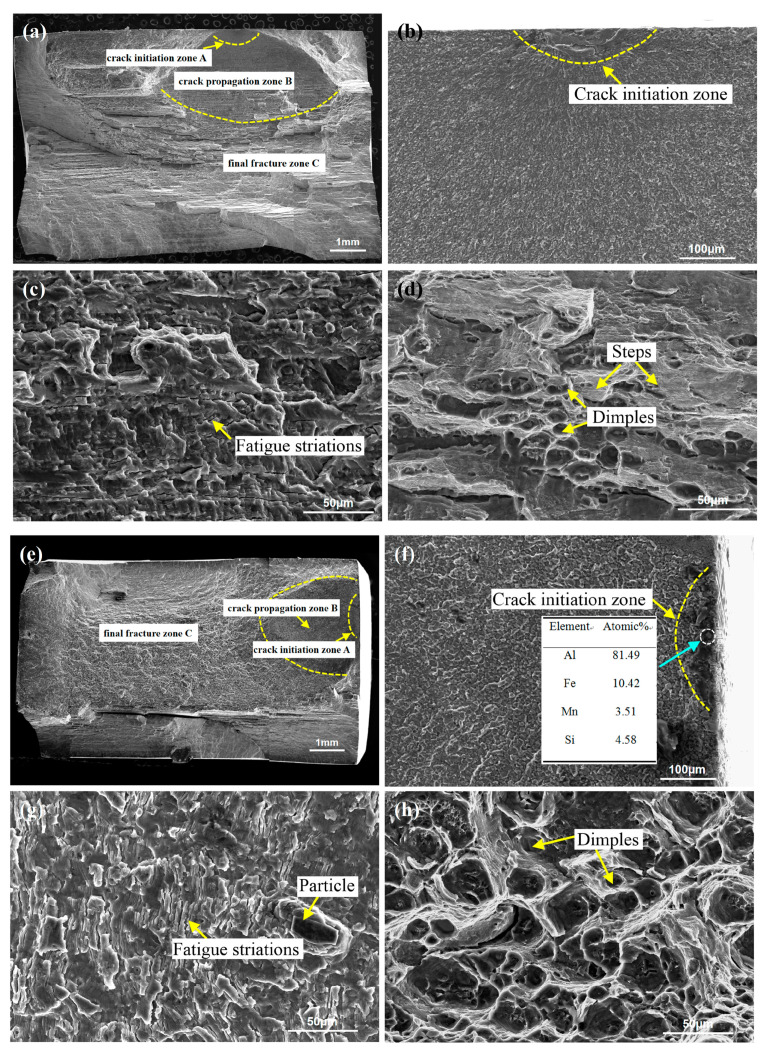
Fracture morphologies of FSW and UAFSW at the maximum stress of 250 MPa: (**a**) FSW macrostructure; (**b**) crack initiation zone from Figure 10a; (**c**) crack propagation zone from Figure 10a; (**d**) final fracture zone from Figure 10a; (**e**) UAFSW macrostructure; (**f**) crack initiation zone from Figure 10(e); (**g**) crack propagation zone from Figure 10e; and (**h**) final fracture zone in Figure 10e.

**Figure 11:**
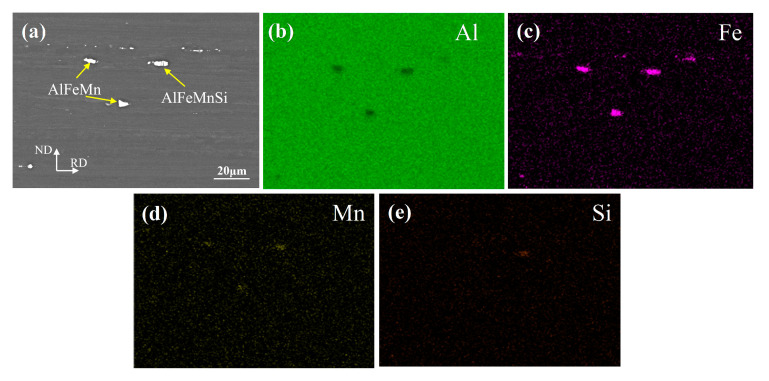
SEM image and elemental mapping results of the 7N01-T4 alloy (**a**); Al element (**b**); Fe element (**c**); Mn element (**d**); Si element (**e**).

**Figure 12:**
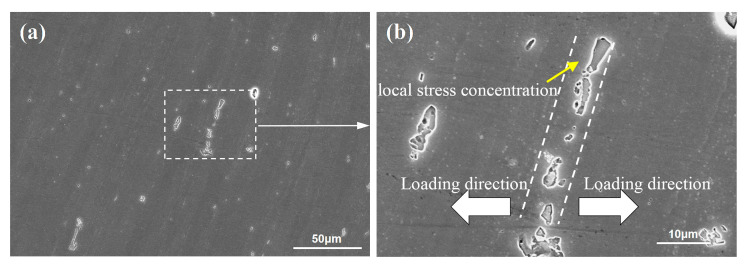
Distribution characteristics of second-phase particles at the FSW joint: (**a**) TMAZ; (**b**) enlargement of (**a**).

Figure 10 reveals the fatigue fracture surface of the FSW and UAFSW specimens under the 250 MPa. The fracture surface can be divided into three regions, namely crack initiation zone (Region A), crack propagation zone (Region B) and final fracture zone (Region C). Figure 10a illustrates that the crack initiation zone is located on the upper surface and characterised by hole features. It is speculated that there are large-sized second-phase particles at this location. The particles and the matrix exhibited an inconsistent collaborative plastic deformation. Hence, the micro-region deformation led to a stress concentration at the particles, which formed micro-cracks (Figure 10b) and eventually led to the falling off of the particles [13]. The crack propagation zone showed a cleavage morphology, and typical fatigue striations could be observed (Figure 10c). Meanwhile, the final fracture zone was distributed with dimples of different sizes and large steps, showing ductile fracture characteristics (Figure 10d). The fatigue fracture of the UAFSW specimen (Figure 10e) indicated that the crack initiation zone occurred near the TMAZ, and the AlFeMnSi particles were found at this position (Figure 10f). In addition to the fatigue striation characteristics, a larger particle was also observed in the crack propagation zone (Figure 10g). Many particles can be found in the 7N01-T4 alloy, namely AlFeMn and AlFeMnSi (Figure 11). Figure 12 shows the second-phase particle distribution at the FSW joint TMAZ. The structural characteristics of the TMAZ bending grains resulted in the particles distribution on the grain boundary becoming approximately perpendicular to the loading direction and being separated from the matrix along the interface under the variable stresses, which led to an easy crack initiation. Therefore, two main causes of the fatigue fracture of the FSW and UAFSW specimens near the TMAZ under high-stress levels were found. First, the values of residual stresses in each micro-area of the joints varied greatly. The large deformation and the high temperature led to relatively large residual stresses at the shoulder edge. In the fatigue test process, when the external and residual stresses superimposition was higher than the yield strength of the micro-zone, plastic deformation occurred at this location and led to fracture. Second, the 7N01-T4 alloy contained many second-phase particles, and the particles on the deformed grain boundaries and matrix were more likely to cause stress concentration and form micro-cracks in the TMAZ.

**Figure 13:**
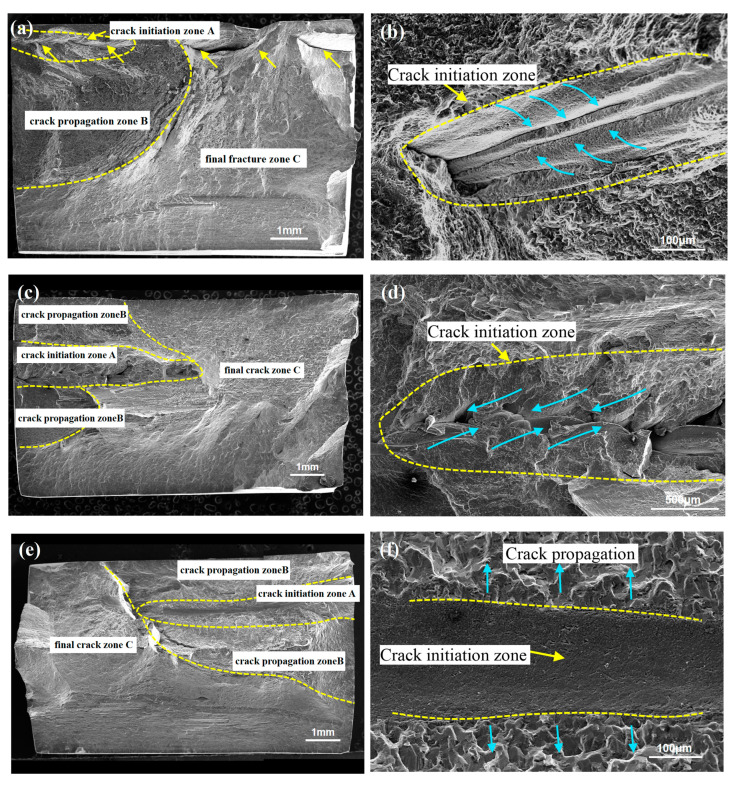
Fracture morphologies of the FSW specimens at the maximum stresses of 230–210 MPa: (**a**) 230 MPa; (**b**) crack initiation zone in Figure 13a; (**c**) 220 MPa; (**d**) crack initiation zone in Figure 13c; (**e**) 210 MPa; and (**f**) crack initiation zone in Figure 13e.

The fatigue cracks of the FSW and UAFSW specimens were initiated from the SZ under low-stress levels. Figure 13 shows the fracture surface of the FSW specimens at 230–210 MPa stress. A clear dividing line was observed at the upper position of the specimen (Figure 13a). The morphological characteristics of the upper and lower metal confluences in the crack initiation zone (blue arrows) can also be clearly observed (Figure 13b). The smooth surface indicated that the metal was less mixed at this location and belonged to the weak bonding region. In addition, a continuous fracture strip (yellow arrows, Figure 13a) was found in the upper part of the joint, which crossed the crack initiation zone, crack propagation zone and final fracture zone, further indicating that the upper part of the specimen was a weak bonding region that resulted in the fatigue crack initiation. Figure 13c shows the fatigue fracture surface of the specimen tested at 220 MPa stress. The upper part of the fracture was characterised by a loose structure, that is, the crack initiation zone. The upper and lower metals overlapped and mixed alternately, which was the last converging position (Figure 13d). The existence of this loose structure indicated that the SZ metal flowed with a time delay, and the two metal chain strands did not converge. Figure 13e shows the fatigue fracture surface of the specimen tested at 210 MPa stress. A boundary strip was also found at the upper position of the fracture, which was a source of cracks. We, however, did not observe if the fatigue initiation was characterised by the metal flow convergence, but it was also the region with a low metal mixing degree (Figure 13e). In summary, under low-stress levels, the crack initiation zone can easily be initiated in the upper part of the FSW fatigue specimens. Accordingly, Wu et al. [26] found similar experimental results, that is, the fatigue cracks can easily be initiated in the weak bond zone near the upper part of the joint.

**Figure 14:**
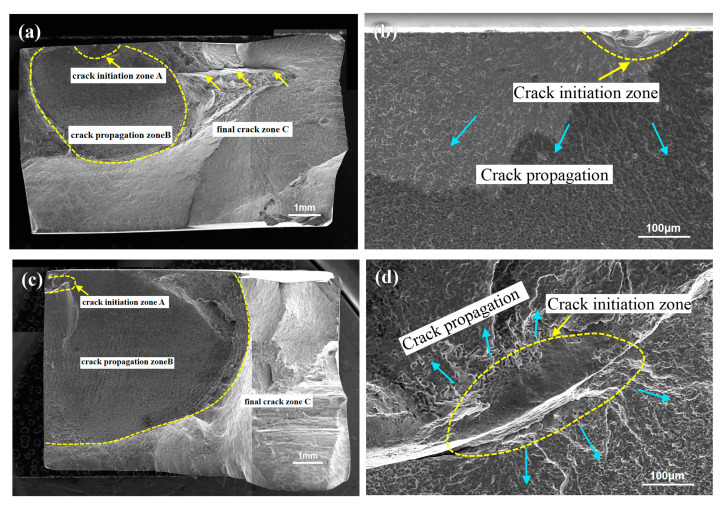
Fracture morphologies of the UAFSW specimens at maximum stresses of 230–225 MPa: (**a**) 230 MPa; (**b**) crack initiation zone from Figure 14a; (**c**) 225 MPa; and (**d**) crack initiation zone from Figure 14c.

Figure 14 shows the fatigue fracture surface of the UAFSW specimens under 230–225 MPa stress. Figure 14a depicts the fracture surface of the specimen tested at 230 MPa stress. The crack initiation zone was located on the top surface (Figure 14b), but a fracture band was observed at the final fracture zone (yellow arrows). The weak connection length of the UAFSW specimen was shorter compared with the FSW (Figure 13a). The fracture morphology of the UAFSW fatigue specimen under 225 MPa stress is illustrated in Figure 14c. The fatigue crack initiation zone was located at the upper part and the fracture was formed by the poor mixing of the upper and lower metals (Figure 14d).

## 4. Discussion

The crack initiation zone of the joint generally appeared in the position of mechanical damage and high stress. In addition, the defects and the complex microstructures in the FSW joints can easily become crack initiation zones. The abovementioned experimental results depicted that under high-stress loading conditions, the specimens are more likely to fracture at the TMAZ-AS. By contrast, under low-stress loading conditions, the specimens are more likely to fracture at the SZ. Generally, the SZ can be simply divided into the shoulder-driven zone (SDZ), pin-driven zone (PDZ), transition zone (TZ) and swirl zone (SWZ) according to the effect of the tool on the surrounding metal [33]. Kissing defects can easily be formed in the SWZ because of the insufficient material flow at the bottom of the joint. In this study, no fatigue fracture occurred at the bottom of the specimen; therefore, the metal flow behaviour of the SDZ, TZ and PDZ during the welding process was mainly discussed.

**Figure 15:**
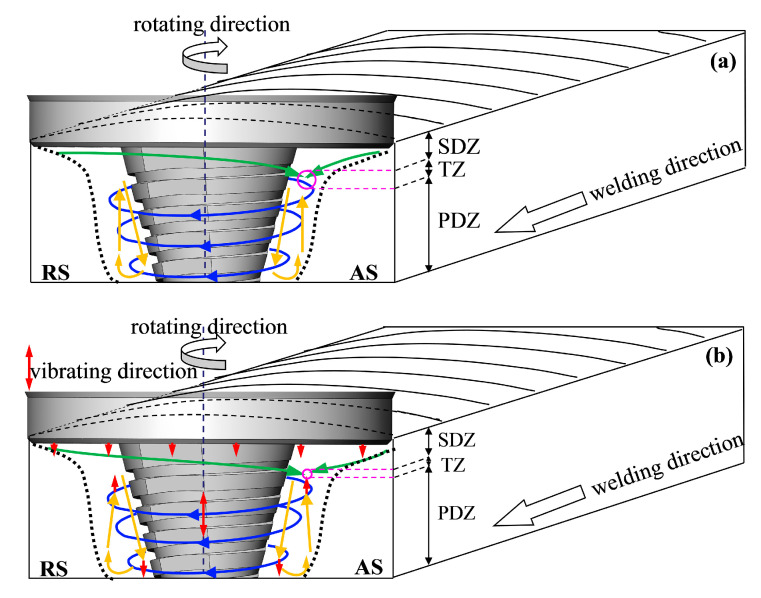
Physical models of the plastic material flow of the SZ in the joints: (**a**) FSW and (**b**) UAFSW.

The observation results of the abovementioned fatigue fracture morphology inferred that cracks in the FSW specimens are easy to initiate at the TZ. It is generally believed that the welding temperature is the main factor that determines the flow behaviour of the SZ, but in the related investigation, the temperature rise caused by ultrasonic vibration is very limited [34]. Thus, when discussing the metal flow behaviour of SZ, the influence of ultrasonic vibration on the temperature is ignored. A further analysis of the metal flow behaviour of the SZ in the FSW process can be decomposed into three modes: (1) Metal rotates circularly; (2) metal moves horizontally; and (3) metal produces the ring vortex flow along the plate thickness [35,36]. In this experiment, the counterclockwise rotation of the right threaded pin drove the material down towards the bottom of the weld [37], and the accumulated metal migrated upward because of the bottom steel plate barrier. The SZ metal flow physical model of the FSW in Figure 15a was established. The SDZ metal was mainly squeezed by the shoulder (green arrows), and the PDZ metal was mainly subjected to the stirring action of the pin (blue arrows). Additionally, the inclination angle of the tool and the thread on the pin drive the metal to produce the ring vortex movement along the plate thickness (yellow arrows). The staggered morphology of the crack initiation zone of the FSW fatigue specimen indicated that the downward flowing metal of the SDZ and the upward migrating metal of the PDZ merged in the TZ. The FSW process exhibited a large temperature gradient along the thickness direction [38], especially at the lower temperature at the bottom of the weld, which led to a poor metal migration ability. The degree of metal mixing between the SDZ and the PDZ was reduced, resulting in a weak bond or a loose defect at the TZ, even holes [39].

The crack initiation zone of the UAFSW specimen was smaller compared with the strip morphology of the continuous crack initiation zone of the FSW specimen (Figure 14c). Combining the morphological characteristics of the crack initiation zone of the UAFSW and the analysis results of the abovementioned SZ metal flow behaviour, we propose herein the SZ metal flow physical model of the UAFSW in Figure 15b. From the macro view of metal flow, applying axial ultrasonic vibration, the high-frequency vibrating shoulder provided an additional forging effect on the SDZ metal and promoted its downward flow. At the same time, the high-frequency vibrating pin also promoted the rapid migration of the PDZ metal in the thickness direction of the plate and reduced the time delay of the SDZ and PDZ metal convergence, thereby resulting in a lower probability of forming a weak bond in the TZ. From the local view of metal flow, the tool and the material were in a state of repeated contact and separation; hence, the superposition of the oscillating and axial stresses of the tool (steady stress) led to a reduction in the average stress and internal strain (stress superposition). The ultrasonic energy introduced into the weld was preferentially absorbed by defects, such as dislocations and grain boundaries in the material, which reduced the maximum shear stress required for the dislocation movement and resulted in a decrease in the material shear stress (acoustic softening) [40]. Shi et al. [41] believed that ultrasonic energy reduced the flow stress and viscosity of the metal near the pin, which led to the increase in the flow velocity and strain rate of the SZ metal. Additionally, simulation results have shown that there is a certain vertical pressure gradient inside the groove of the pin during the FSW process, which drives the metal in the pin thread to flow downward [42]. The periodically changing pressure gradient in the groove was perhaps caused by the axial ultrasonic vibration, and the metal flowed more intensely. In conclusion, the application of axial ultrasonic vibration not only drives the acceleration and convergence of the SDZ and PDZ metals by the shoulder and the pin, but also reduces the flow stress of the SZ metal because of the effect of stress superposition and acoustic softening and the metal flow of SZ being more sufficient.

## 5. Conclusions

This study performed an axial ultrasonic vibration-assisted friction stir welding of the 7N01-T4 alloy and investigated the microstructure characteristics and residual stresses distribution of the FSW and UAFSW joints. Furthermore, the effects of the axial ultrasonic vibration on the fatigue properties and fatigue fracture behaviour were also discussed. The following conclusions are drawn from this study:

(1) The application of axial ultrasonic vibration can promote the metal flow of the SZ, resulting in a greater deformation of the SZ and an increased strain rate of the micro-regions for refining grains. Using the UAFSW method can significantly improve the fatigue performance of the welded joint. The fatigue limit of the UAFSW specimen is 220 MPa, which is 20 MPa higher than that of the FSW.
(2) The difference in the transverse residual stresses between the FSW and the UAFSW is very small. The maximum longitudinal residual stresses of the two joints is located near the edge of the tool shoulder on the AS, which can be reduced by 17.8% by ultrasonic vibration. This may be mainly due to the reduction in the boundary distortion of SZ/TMAZ and the local heat generation by ultrasonic vibration.
(3) The joints show different fracture modes under different stress conditions. Both FSW and UAFSW specimens fracture at the TMAZ-AS under high-stress levels. Under alternating loads, second-phase particles on the TMAZ grain boundary are likely to cause stress concentration. Moreover, micro-zone high residual stresses and SZ/TMAZ microstructure difference also aggravate the nonuniform deformation, leading to crack initiation.
(4) Under low-stress levels, fatigue crack initiation is prone to occur near the TZ and eventually fracture in the SZ. Axial ultrasonic vibration promotes the metal convergence from the SDZ and the PDZ, resulting in a more uniform and denser microstructure of TZ, thereby reducing the probability of forming loose defects in the TZ and improving the fatigue life.
